# IL-37/ STAT3/ HIF-1α negative feedback signaling drives gemcitabine resistance in pancreatic cancer

**DOI:** 10.7150/thno.42416

**Published:** 2020-03-04

**Authors:** Tiansuo Zhao, Fanjie Jin, Di Xiao, Hongwei Wang, Chongbiao Huang, Xiuchao Wang, Song Gao, Jing Liu, Shengyu Yang, Jihui Hao

**Affiliations:** 1 Tianjin Medical University Cancer Institute and Hospital, National Clinical Research Center for Cancer, Key Laboratory of Cancer Prevention and Therapy, Tianjin's Clinical Research Center for Cancer, Department of Pancreatic Cancer, Tianjin, PR China.; 2 Department of Cellular and Molecular Physiology, Penn State College of Medicine, Hershey, PA.

**Keywords:** Pancreatic ductal adenocarcinoma, IL-37, Hypoxia-inducible factor (HIF) -1α, Gemcitabine resistance

## Abstract

Human interleukin (IL)-37 is a member of the IL-1 family with potent anti-inflammatory and immunosuppressive properties. Previously, it has been reported that IL-37 suppresses tumor growth and progression. However, the roles of IL-37 in pancreatic cancer development and chemo-resistance remain unknown.

**Methods**: Immunohistochemistry was used to analyze the correlation between IL-37 expression and clinicopathological features of pancreatic ductal adenocarcinoma (PDAC). Western-blot and RT-PCR was used to verify the correlation between IL-37 and hypoxia-inducible factor (HIF)-1α. We performed chromatin immunoprecipitation and luciferase assays to validate HIF-1α suppression of IL-37 expression. Moreover, gain- and loss-of-function studies *in vitro* and *in vivo* were used to demonstrate the biological function of IL-37 on PDAC development and chemo-resistance.

**Results**: Our results showed that IL-37 expression was remarkably decreased in PDAC tissues when compared to adjacent normal pancreatic tissues. Reduced IL-37 expression in PDACs was associated with increased PDAC histological grade, tumor size, lymph node metastasis and vessel invasion. IL-37 low patients also have remarkably shorter relapse-free and overall survival. Importantly, IL-37 expression was positively correlated with Gemcitabine efficacy. Mechanistically, HIF-1α attenuated IL-37 transcription by binding to the hypoxia response elements (HREs) in IL-37 promoter. Conversely, IL-37 suppressed HIF-1α expression through STAT3 inhibition. Functionally, downregulation of IL-37 in PDAC cells promoted chemo-resistance, migration and progression *in vivo* and *in vitro*.

**Conclusions**: Collectively, our data uncovered IL-37/ STAT3/ HIF-1α negative feedback signaling drives Gemcitabine resistance in PDAC.

## Introduction

Interleukin-37 (IL-37; formerly IL-1 family member 7) has been identified as an inhibitor of both innate and adaptive immunity 1,2. IL-37 has five splice transcript variants that encode distinct isoforms (IL-37a-IL-37e) 3,4. IL-37b is the largest isotype that has 5 of the 6 exons. IL-37b has biologically functional and can produce homodimers 5. Other IL-37 isoforms are either not functional, or their function is undetermined.

Over recent years, the anti-inflammatory functions of IL-37 have been extensively studied. IL-37 suppresses production of pro-inflammatory cytokines 1 and the maturation of dendritic cells 6. There is also evidence that IL-37 can translocate into the nucleus in a caspase-1-dependent manner to decrease cytokine production and affect innate and adaptive immune responses 7,8.

Previously, there have been reports suggesting that IL-37 suppresses tumor growth and progression in fibrosarcoma 9, human hepatocellular carcinoma (HCC) 10,11 and lung cancer 12. Intracellular mature IL-37 (not its extracellular form) was able to suppress lung cancer metastasis via inhibiting Rac1 13. It has been reported that intracellular IL-37b can interact with Smad3 to suppress multiple signaling pathways (such as ERK, p38 MAPK, JNK, PI3K, NF-κB, and STAT3 pathways) and modulate the expression of metastasis-related genes in tumor cells 14. However, the biological functions and the exact molecular mechanisms of IL-37 in pancreatic ductal adenocarcinoma (PDAC) development and chemo-resistance are yet to be examined.

PDAC is the fourth leading cause of cancer-related death in the United States, with a dismal 5-year survival rate of no more than 9% 15. Despite recent development in PDAC diagnosis and therapy, there is still little improvement at the survival rate. PDAC is characterized by an abundant desmoplastic stroma fibrosis and the desmoplasia causes hypo-vascularity and poor blood supply resulted in a hypoxic milieu in pancreatic cancer 16. Gemcitabine (Gem), as the standard treatment for PDAC, has been developed resistance in most of the treated patients due to desmoplastic stroma and abnormal signaling pathways 17. PDAC cells constitutively express hypoxia-inducible factor (HIF)-1α in the hypoxic microenvironment 18 and HIF-1α, as a nuclear factor, can bind to the hypoxia response elements (HREs) of target genes and regulate their transcription 19. MUC1 and HIF-1α signaling crosstalk induces anabolic glucose metabolism and imparts Gem resistance to PDAC 20. So, whether IL-37 influences Gem efficacy by HIF-1α signaling is still unknown.

In the present study, we explored the functions and molecular mechanisms of IL-37 in PDAC progression and chemo-resistance. Our data demonstrated that PDAC patients have lower IL-37 level in serum than healthy people. The IL-37 expression also decreased in PDAC tissues and reduced IL-37 expression was closely associated with clinicopathological features. Importantly, IL-37 expression was positively correlated with Gem efficacy. Mechanistically, HIF-1α attenuated IL-37 transcription by binding to the HREs in IL-37 promoter. Conversely, IL-37 suppressed HIF-1α expression through STAT3 inhibition. Functionally, downregulation of IL-37 in PDAC cells promoted chemo-resistance, motility and migration *in vivo* and *in vitro*. Collectively, our data uncovered IL-37/STAT3/HIF-1α negative feedback signaling drives Gem resistance in PDAC.

## Materials and methods

### Enzyme-linked immunoassay (ELISA)

We recruited 42 PDAC patients from Tianjin Medical University Cancer Institute and Hospital and 30 healthy donors who were matched with the patients regarding to the age and gender. Peripheral blood samples (3-5 ml) were collected. Serum was prepared through centrifugation and stored at -80 °C. Measurement IL-37 levels in serum were performed using ELISA according to the manufacturer's instructions (R&D Systems, Minneapolis, USA). The protocol of study was approved by Ethical Committee on Human Research. Informed written consent was achieved from patients.

### Immunohistochemistry (IHC)

With approval from the Ethics Committee, PDAC samples were obtained from 85 patients (aged 31 years to 73 years) undergoing surgical resection with histologic diagnosis of PDAC at the Tianjin Medical University Cancer Institute and Hospital. Another cohort was 76 PDAC patients undergoing surgical resection and Gem treatment at the Tianjin Medical University Cancer Institute and Hospital. Immunohistochemistry for IL-37, HIF-1α and p-STAT3 (705) of PDAC patient tissues was performed using a DAB substrate kit (ZSGB-BIO, Beijing, China). Immunoreactivity was semi-quantitatively scored according to the estimated percentage of positive tumor cells as previously described 21. Staining intensity was scored 0 (negative), 1 (low), 2 (medium), and 3 (high). Staining extent was scored 0 (0% stained), 1 (1%-25% stained), 2 (26%-50% stained), and 3 (51%-100% stained). The final score was determined by multiplying the intensity scores with staining extent and ranged from 0 to 9. Final scores (intensity score × percentage score) less than 2 were considered as negative staining (-), 2-3 were low staining (+), 4-6 were medium staining (++) and > 6 were high staining (+++).

### Cell culture and hypoxic treatment

Human PDAC cell lines, PANC-1, SW1990, BxPC-3, CFPAC-1 and HEK 293 were obtained from the Committee of Type Culture Collection of Chinese Academy of Sciences (Shanghai, China) and MIA-PaCa2 was obtained from the American Type Culture Collection. The Gem-resistance PDAC cell lines (FG and BxPC-3) were a gift from Prof. Keping Xie (MD Anderson Cancer Center, Houston, TX). All the cell lines were obtained in 2013, and recently authenticated in August 2018 through the short tandem repeat analysis method. These cells were grown at 37 °C in a humidified atmosphere of 95% air and 5% CO_2_ using Dulbecco's modified Eagle medium (DMEM) with 10% fetal bovine serum (FBS). For hypoxic treatment, cells were placed in a modulator incubator (Thermo Electron Co., Forma, MA, USA) in an atmosphere consisting of 93.5% N_2_, 5% CO_2_, and 1.5% O_2_.

### Western blot analysis

Whole-cell extracts were prepared by lysing cells with RIPA lysis buffer supplemented with a proteinase inhibitor cocktail (Sigma). Protein concentrations were quantified using Pierce protein assay kit (Pierce). Protein lysates (20 μg) were separated by SDS-PAGE, and target proteins were detected by Western blot analysis with antibodies (Table S1). Specific proteins were visualized using an enhanced chemiluminescence detection reagent (Pierce).

### Reverse-transcription polymerase chain reaction (RT-PCR)

Total RNA was isolated from transfected cells with TRIzol Reagent (Invitrogen) and used for first-strand cDNA synthesis using the First-Strand Synthesis System for RT-PCR (Takara). Each sample was processed in triplicate, and β-actin was used as loading control. Each experiment was repeated independently for at least three times. PCR primers used are indicated in Table S1.

### Chromatin immunoprecipitation assay (ChIP)

Chromatin immunoprecipitation assay was performed using a commercial kit (Upstate Biotechnology) according to the manufacturer's instructions 19, 21. The PCR primers are indicated in Table S1.

### siRNA duplexes, plasmid constructs, transient transfection, stable transfection in pancreatic cancer cells and luciferase assay

Small interfering RNAs (siRNAs) again siIL-37, siIL-18Rα, siIL-1R8 and siHIF-1α were designed and synthesized from GenePharma (Shanghai, China) (Table S1). The human IL-37 and HIF-1α cDNA was cloned into the pLV plasmid expression vector.

IL-37 overexpression in PDAC cell lines, Lentivirus-mediated plasmid was done using the pLV-cDNA system (Biosettia) following the manufacturer's instructions. Lentivirus encoding DNA was packaged as previously described 21. Following transfection, the medium containing Lentivirus was collected, filtered, and transferred onto PDAC cell lines. Infected cells were selected with puromycin (1 μg/mL) for 7 days.

Genomic DNA fragments of the human IL-37 gene, spanning from +1 to -2000 relative to the transcription initiation sites were generated by PCR and inserted into pGL3-Basic vectors (denoted as pGL3-IL37). All constructs were sequenced to confirm their identity. Luciferase activity was measured using the Dual-Luciferase Reporter Assay System (Promega) as previously described 21. For transfection, cells were plated at a density of 5×10^5^ cells/well in 6-well plates with serum-containing medium. When the cells were 80% confluent, the siRNA duplexes or overexpression plasmids were transfected into cells using Lipofectamine-2000 (Invitrogen) for 48 h. The cells were collected for cell motility and migration analysis, Western blot analysis, and RT-PCR, etc.

### Cell cycle and apoptosis assay

PANC-1, MIA-PaCa2 and SW1990 cell lines were treated with rIL-37 (100 ng/ml), Gem (2 μM) and Gem plus rIL-37 for 24 h and divided into four groups. Prior to the treatment, cells were cultured overnight in serum-free conditions to synchronize cell growth. Cells were trypsinized and fixed in cold 70% ethanol for 10 min and then stained with propidium iodide (PI) solution at room temperature for 15 min. Approximately 10,000 sample cells were analyzed using flow cytometer (Beckman Coulter) with excitation at 488 nm and emission at 617 nm. The percentage of cells in each phase of the cell cycle was determined using CXP System Software (Beckman Coulter).

PANC-1, MIA-PaCa2 and SW1990 cell lines treated with rIL-37 or/and Gem were analyzed for phosphatidylserine exposure by an annexin-V FITC/PI double-staining method using a commercial kit (BD Biosciences, Cat. 556570) according to the manufacturer's instructions. A minimum of 5,000 cells were then analyzed by FACScan with Cell Quest software (Beckton Dickinson) for acquisition and analysis.

### Wound healing and Cell migration assay

A wound healing assay was performed according to published protocol 22. Migration assays were performed with 8.0 μm pore inserts in a 24-well Transwell. For this assay, 1×10^5^ cells were isolated and added to the upper chamber of a transwell with DMEM. DMEM with 10% fetal bovine serum was added to the lower chamber and the cells incubated for 18 h. Cells that had migrated to the bottom of the filter were stained with a three-step stain set (Thermo Scientific). All experiments were repeated independently for at least three times.

### Animal studies in subcutaneous pancreatic cancer mouse model

Female 4-week-old nude nu/nu mice were maintained in a barrier facility on HEPA-filtered racks. All animal studies were conducted under an approved protocol in accordance with the principles and procedures outlined in the NIH Guide for the Care and Use of Laboratory Animals. Recombinant human IL-37 (rIL-37) was purchased from PeproTech Company. The Gem was obtained from Tianjin Medical University Cancer Institute and Hospital. Cells were harvested by trypsinization, washed in PBS, resuspended at 10^7^ cells/ ml in a 1:1 solution of PBS/Matrigel, and injected subcutaneously into two flanks of 5 nude nu/nu mice (10^6^ cells). Seven days after tumor xenograft, we divided 20 nude nu/nu mice into four groups (Saline, rIL-37, Gem and Gem plus rIL-37, respectively, 5 mice each) and intraperitoneal injected with Saline, rIL-37 (100 ng/g) 23, Gem (50 mg/kg) and Gem plus rIL-37 every three days. Primary tumors were measured in 3 dimensions (a, b, c), and volume was calculated as abc×0.52 21. After 35 days primary tumors were harvested from the flanks of nude nu/nu mice. The tumor was fixated by formalin and embedded using paraffin for IHC analysis.

### Statistical analysis

Student's t-test for paired data was used to compare mean values. ANOVA is used to analysis two groups' data with continuous variables. Non-parametric data were analyzed with Mann-Whitney U test. The categorical data was analyzed by either Fisher's exact or Chi-Square method. Each experiment was conducted independently for at least three times, and values were presented as mean ± standard deviation (SD), unless otherwise stated. Analyses were performed using SPSS21.0 statistical analysis software.

## Results

### IL-37 secretion and expression was decreased in serum and PDAC specimens

**Figure 1:**
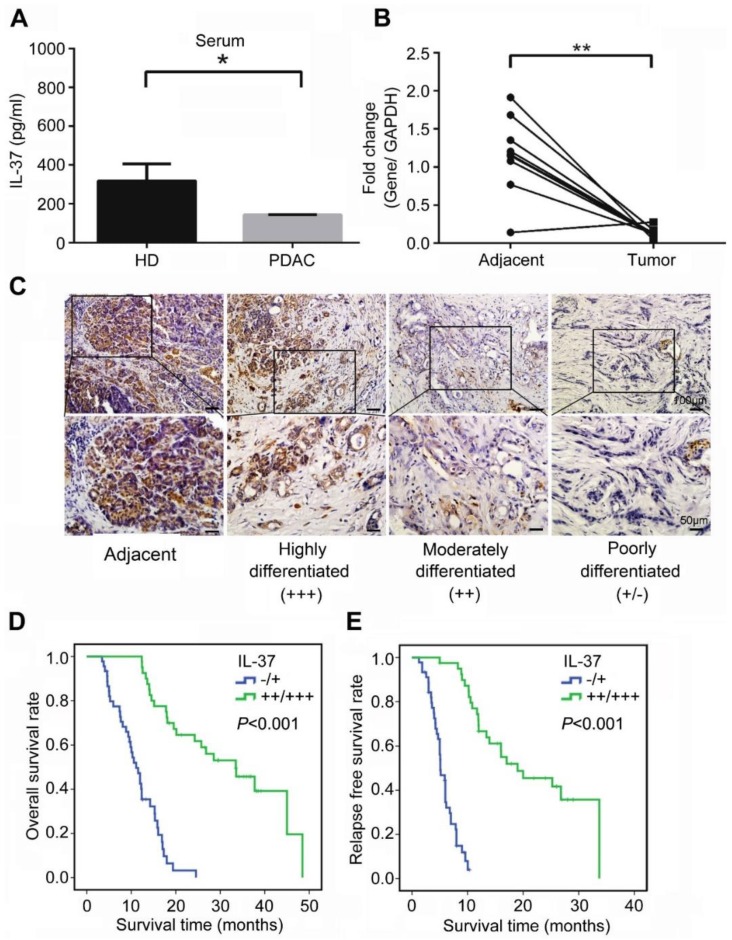
** IL-37 reduced expression in PDACs indicated poor survival durations. (A)** IL-37 secretion level in serum of PDACs and paired health peoples examined by ELISA kit. **(B)** Quantitative reverse transcription-PCR analysis of IL-37 mRNA level in 10 paired PDAC specimens. **(C)** Immunohistochemical analysis of IL-37 protein expression in TMA of PDAC and adjacent normal pancreatic tissues. **(D)** Association of IL-37 expression with OS rate in patients with PDAC. Patients with low IL-37 expression (intensity grade, -/+) had much shorter OS durations than did patients with high IL-37 expression (intensity grade, ++/+++; *P* < 0.001 [log-rank test]). **(E)** Association of IL-37 expression with RFS rate in PDAC patients (*P* < 0.001 [log-rank test]). *P < 0.05 and **P < 0.01.

We first examined the level of serum IL-37 in PDAC patients and age and gender -matched healthy donors. The result showed that serum IL-37 level was significantly reduced in PDAC patients when compared with healthy donors (Figure 1A, P < 0.05). Next, we used 10 paired PDAC specimens including tumor tissues and adjacent tissues to examine the expression of IL-37 mRNA. Our data indicated that the IL-37 mRNA was also remarkably decreased in tumor tissues when compared with adjacent tissues (Figure 1B, P < 0.01). Through IHC staining of tissue microarray (TMA) containing PDAC and adjacent normal pancreatic tissues, we found IL-37 protein expression was notably lower in PDACs than in normal tissues and IL-37 expression was positively correlated with the differentiation degrees of PDAC (Figure 1C), indicating that IL-37 expression was reduced during PDAC progression.

### IL-37 expression was associated with overall and relapse-free survival in PDACs

To investigate the pathologic significance of IL-37 expression in PDAC progression, we analyzed the correlation between IL-37 expression and clinicopathological features of PDAC (Table 1). There was no correlation between IL-37 expression and age and gender among PDAC patients. However, IL-37 expression was negatively correlated with histologic grade (χ^2^ = 26.972, *P* < 0.01, *r* = -0.563), tumor size (χ^2^ = 18.378, *P* < 0.01, *r* = -0.465), lymph node metastasis (χ^2^ = 39.178, *P* < 0.01, *r* = -0.679), and vessel invasion (χ^2^ = 19.552, *P* < 0.01, *r* = -0.480) (Table 1). Importantly, Kaplan-Meier analysis of TMA data indicated that PDAC patients with negative (-) or low (+) IL-37 protein expression had significantly worse median overall survival (OS) and relapse-free survival (RFS) than those with moderate (++) or high (+++) IL-37 protein expression (P < 0.001; OS: 11.1 and 33.5 months, respectively; RFS: 5.1 and 19.0 months, respectively) (Figure 1D-E). We then performed univariate and multivariate analysis of clinical follow-up data of PDAC patients (Table 2). Intriguingly, IL-37 expression was positively correlated with both OS and RFS in univariate and multivariate analyses. These data indicated that loss of IL-37 expression in PDAC was an independent risk factor for PDAC progression.

### IL-37 expression was positively correlated with Gem efficacy and negatively correlated with HIF-1α expression in PDAC

**Figure 2:**
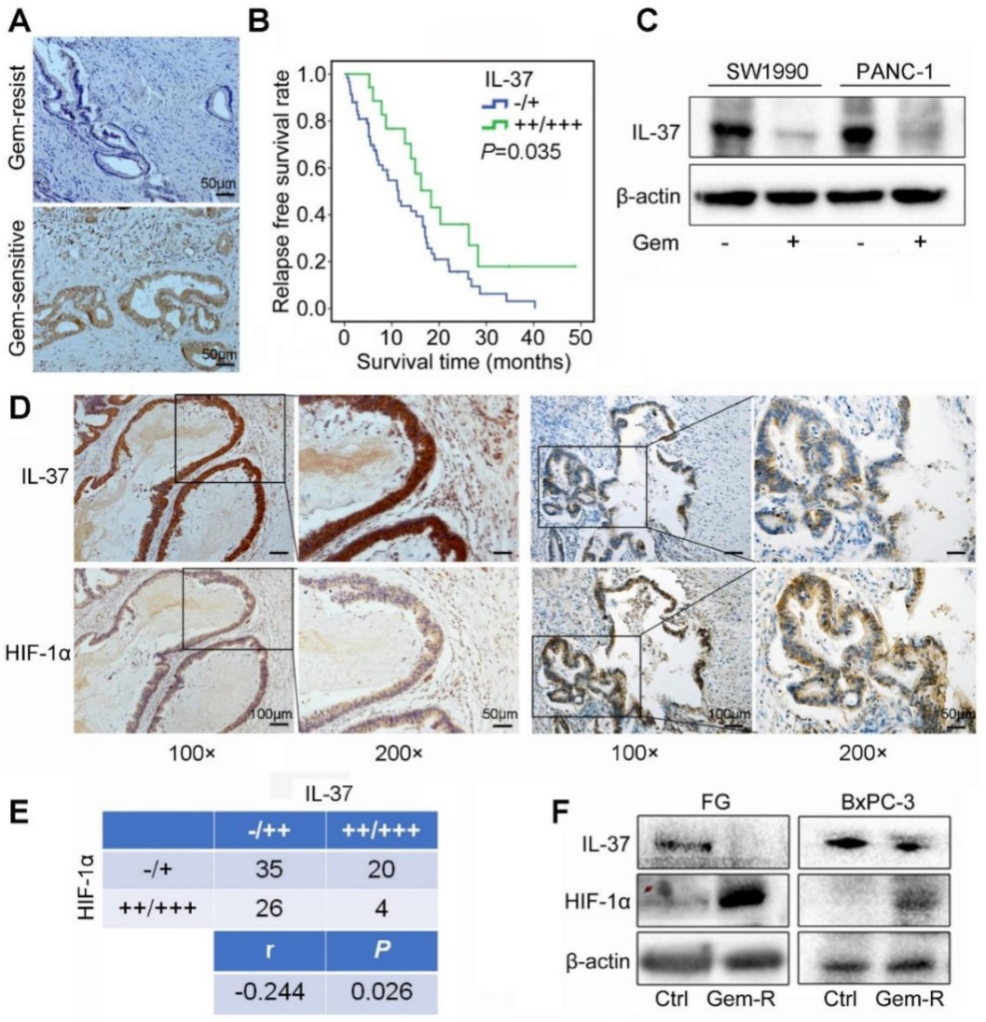
** IL-37 expression was positively correlated with Gem efficacy and negatively correlated with HIF-1α expression in PDAC. (A)** Representative images for immunohistochemical IL-37 staining in Gem-resist and sensitive PDAC patients. **(B)** Association of IL-37 expression with RFS rate in PDAC patients with Gem treatment (n=76, *P* = 0.035). **(C)** Western blot analysis of IL-37 expression in SW1990 and PANC-1 cells treated with the Gem (2 µM) for 48 h. **(D) and (E)** IHC assay of the expression of HIF-1α and IL-37 in human PDAC samples (*n* = 85, *P* = 0.026). **(F)** Western blot analysis of IL-37 and HIF-1α expression in Gem-resistance cells (FG and BxPC-3).

To investigate the correlation between IL-37 expression and Gem efficacy, we analyzed the IHC data from PDAC patients treated with Gem as adjuvant chemotherapy. It showed that IL-37 protein expression was notably lower in Gem-resist group than in Gem-sensitive group (Figure 2A). Patients with lower IL-37 expression in primary tumors had a significantly decreased RFS (P = 0.035) (Figure 2B).

Aforementioned data indicated that IL-37 played a crucial role in Gem resistance. Then, we treated SW1990 and PANC-1 cell lines with Gem and found IL-37 expression to be decreased with the Gem treatment (Figure 2C).

To determine the relationship between HIF-1α and IL-37 in PDAC, we investigated the expression of HIF-1α and IL-37 in human PDAC samples by IHC staining. As shown in Figure 2D, there was a negative relationship between HIF-1α and IL-37 expression in consecutive sections of PDACs. Importantly, the statistical data confirmed the negative relationship between HIF-1α and IL-37 expression (P = 0.026, r = -0.244) (Figure 2E). Next, we examined IL-37 and HIF-1α expression in Gem-resistance cells (FG and BxPC-3). The results showed that IL-37 expression was down-regulated, but HIF-1α expression was up-regulated in Gem-resistance cell lines (Figure 2F). Desmoplasia is major contributor for Gem resistance. Whether IL-37 can affect the desmoplasia of PDAC is unknown. We tested IL-37 and α-SMA expression in PDAC samples by IHC and performed the Masson stain (n=85). It showed that IL-37 was not associated with α-SMA expression and stromal fibrosis (Figure S1, P > 0.05).

Taken together, these data suggested that IL-37 decreased expression involved in the Gem resistance and IL-37 and HIF-1α expression was negatively correlated in PDAC.

### HIF-1α suppressed the expression of IL-37 in PDAC by binding to the HRE of the IL-37 gene promoter

**Figure 3:**
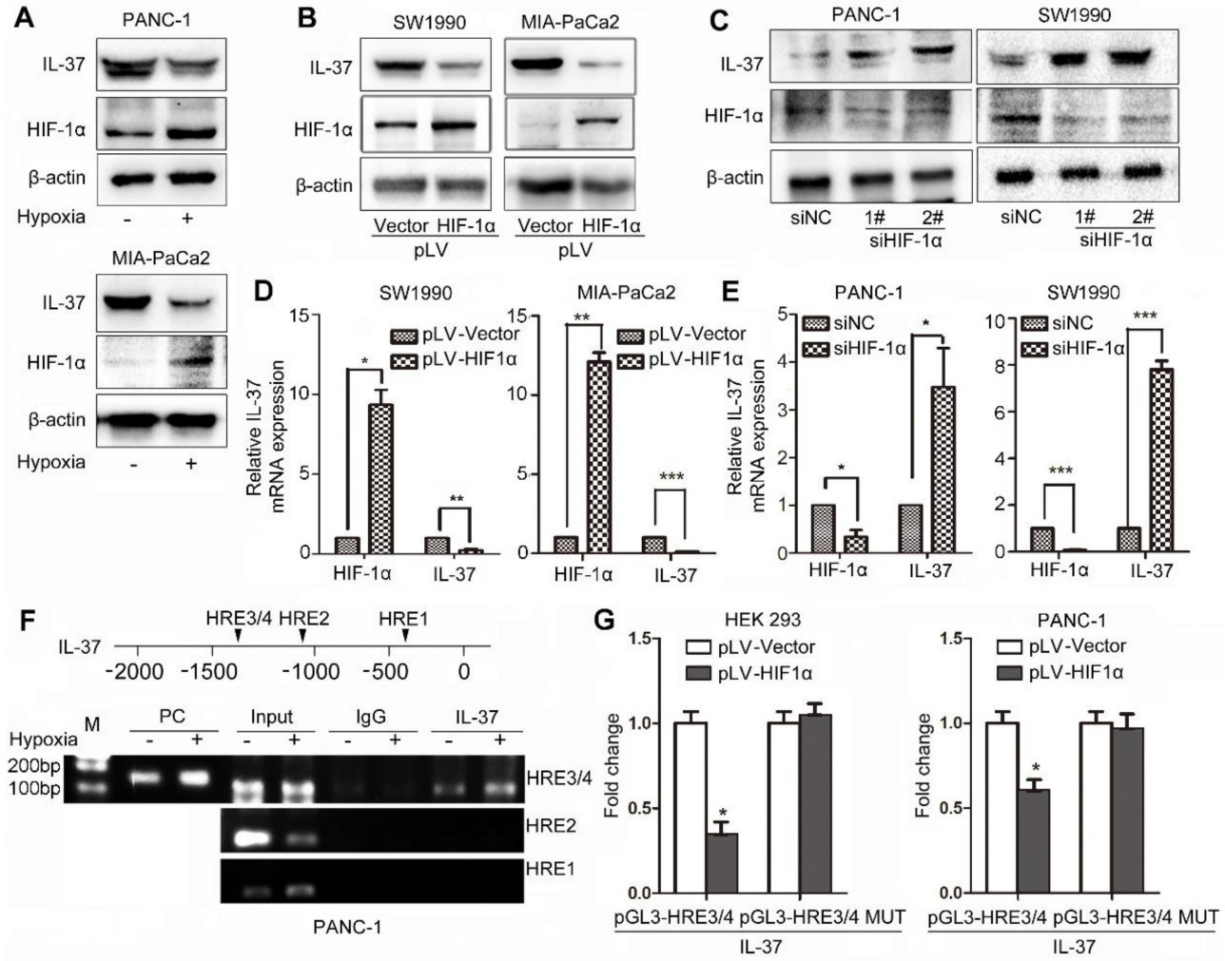
** HIF-1α suppressed the expression of IL-37 in PDAC by binding to the HRE of the IL-37 gene promoter. (A)** Western blot analysis of IL-37 and HIF-1α expression in PANC-1 and MIA-PaCa2 cell lines cultured in hypoxia milieu. Western blot **(B)** and RT-PCR **(D)** analysis of IL-37 and HIF-1α expression in SW1990 and MIA-PaCa2 cell lines transfected with pLV-HIF-1α (1.5 µg). Western blot **(C)** and RT-PCR **(E)** analysis of IL-37 and HIF-1α expression in PANC-1 and SW1990 cell lines transfected with siHIF-1α (50 nM). **(F)** ChIP analysis of HIF-1α binding to the IL-37 promoter in PANC-1 cells under hypoxia treatment. **(G)** Dual-luciferase assay-based promoter activity analysis of HEK293 and PANC-1 cells overexpressing HIF-1α (pLV-HIF1α) and control cells transfected with pGL3-HRE3/4 and pGL3-HRE3/4 mutation plasmids. The cells were subjected to dual luciferase analysis after 48 h transfection. The results are expressed as fold induction relative to that in corresponding cells transfected with the control vector after normalization of firefly luciferase activity according to Renilla luciferase activity. The data are expressed as the means ± SD from three independent experiments. *P < 0.05, **P < 0.01 and ***P < 0.001.

To identify whether HIF-1α can influence IL-37 expression, we treated PDAC cell lines with hypoxia culture and transfected cells with pLV-HIF-1α and siHIF-1α. We found that IL-37 expression was reduced in PANC-1 and MIA-PaCa2 cell lines after hypoxia treatment for 12 h (Figure 3A). Furthermore, IL-37 expression was downregulated by ectopically expressed HIF-1α at protein and mRNA levels (Figure 3B-D). Conversely, HIF-1α knockdown increased the protein and mRNA levels of IL-37, suggesting that HIF-1α inversely regulated IL-37 expression (Figure 3C-E).

We examined the promoter region of the human IL-37 gene and identified four HREs, suggesting that HIF-1α might directly regulate the transcription of IL-37 (Figure 3F upper). To determine whether HIF-1α directly binds to the IL-37 promoter, we performed a ChIP assay using the PANC-1 cell line. In chromatin fractions pulled down by an anti-HIF-1α antibody, we detected HRE3/4 in the IL-37 promoter (Figure 3F down). The fraction immunoprecipitated by the anti-HIF-1α antibody increased significantly (P < 0.01) at HRE3/4 with hypoxia treatment, suggesting that HIF-1α expression influenced the HIF-1α binding to the IL-37 promoter.

To determine whether binding of HIF-1α to the IL-37 promoter activates the promoter, we constructed IL-37 luciferase promoter vectors-pGL3-HRE3/4 and pGL3-HRE3/4 mutation and transfected them with or without a HIF-1α expression vector (pLV-HIF-1α) into HEK293 and PANC-1 cells. Dual-luciferase analysis demonstrated that HIF-1α overexpression significantly suppressed IL-37 promoter activity in HEK293 and PANC-1 cell lines (Figure 3G, P < 0.05). Taken together, these results suggested that HIF-1α can not only directly bind to the IL-37 promoter but also suppress the transcription of IL-37.

### IL-37 sensitized PDAC cells to Gem *in vivo*

**Figure 4:**
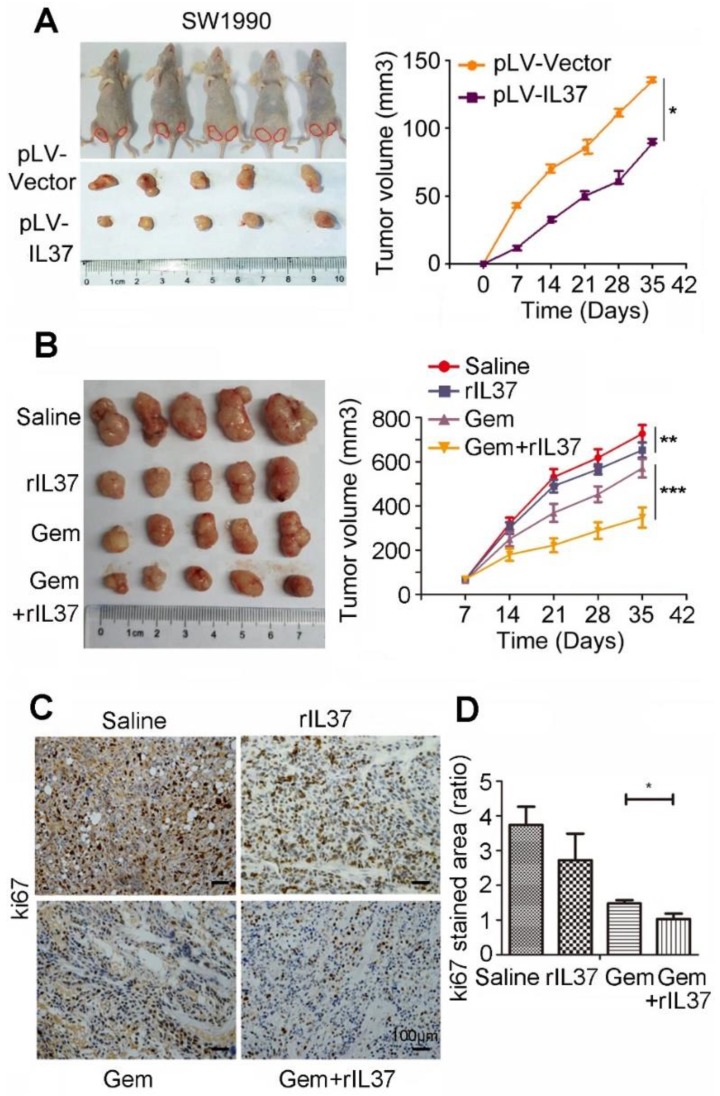
** IL-37 sensitized PDAC cells to Gem *in vivo*. (A)** Subcutaneous pancreatic cancer mouse model using SW1990/pLV-Vector and SW1990/pLV-IL37 cells (*n* = 5/group). Left, shown are representative images of tumors in nude mice (nu/nu); Right, statistical analysis of the volume about all primary tumors. The data are presented as the means ± SD. **(B)** Subcutaneous pancreatic cancer mouse models were intraperitoneally injected with saline, rhIL-37, Gem and Gem plus rhIL-37. **(C)** IHC assays of ki67 expression in the mouse PDAC samples with different treatment. **(D)** The ratio of stained area on ki-67 in mouse samples. *P < 0.05, **P < 0.01 and ***P < 0.001.

In order to test the function of IL-37 in Gem treatment *in vivo*, we developed a subcutaneous pancreatic cancer mouse model using SW1990/pLV-Vector and SW1990/pLV-IL37 cells. When compared tumor volume with SW1990/pLV-Vector cells, the tumor volume of SW1990/pLV-IL37 cells became smaller, suggesting the suppression of IL-37 on PDAC cells (Figure 4A, P < 0.05). Next, we injected SW1990 cells subcutaneously into the right flank of nude nu/nu mice. The mice were intraperitoneally injected with saline, rhIL-37 (100 ng/g), Gem (50 mg/kg) and Gem plus rhIL-37. Compared with the saline control group, the average tumor volume in the rhIL-37 and Gem group was reduced (P < 0.01). Importantly, there was an obviously tumor shrink in Gem plus rhIL-37 group (Figure 4B, P < 0.001). And we performed the IHC assays with the mouse PDAC samples and it showed ki67 expression were extremely inhibited in Gem plus rhIL-37 group compared other groups (Figure 4C-D, P < 0.05). Taken together, our data supported that IL-37 sensitized PDAC cells to Gem *in vivo*.

### IL-37 increased GEM efficacy and inhibited PDAC cells migration *in vitro*

**Figure 5:**
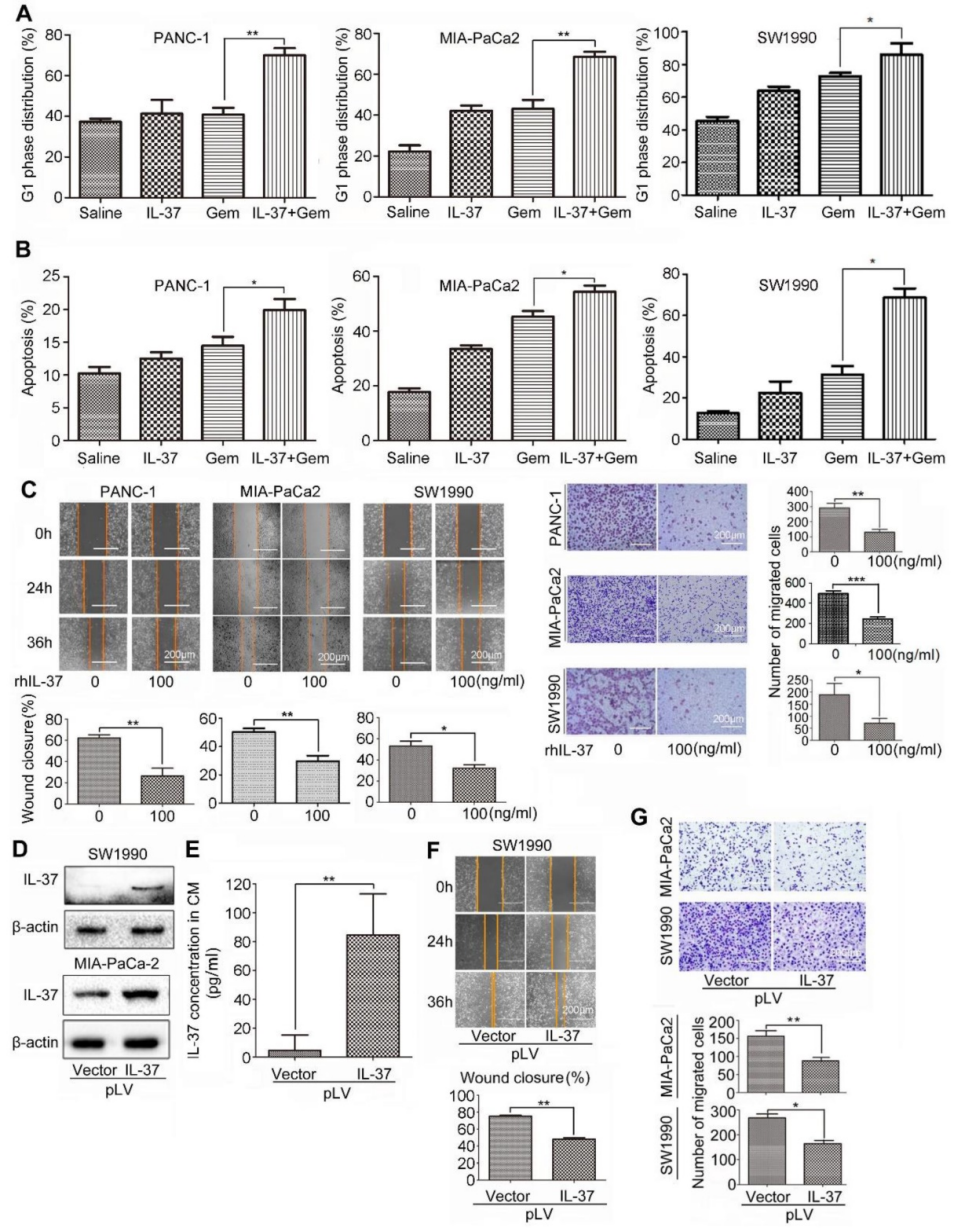
** IL-37 increased GEM efficacy and inhibited PDAC cells migration *in vitro*.** Flow cytometry to analyze the G1 phase distribution **(A)** and apoptosis **(B)** of PANC-1, MIA-PaCa2 and SW1990 cell lines treated with rhIL-37 (100 ng/ml) and Gem (2 µM) for 24 h. **(C)** Left, wound-healing assays comparing the motility of PANC-1, MIA-PaCa2 and SW1990 cell lines treated with rhIL-37 protein for 24 h and 36 h. Right, comparison of the migration of PANC-1, MIA-PaCa2 and SW1990 cell lines treated with rhIL-37 protein for 18 h. **(D)** Western blot analysis of IL-37 expression in SW1990 and MIA-PaCa2 cell lines transfected with pLV-IL37 plasmid (2 µg). **(E)** The secretion of IL-37 in culture supernatant with transfected cells examined by ELISA kit. Wound-healing **(F)** and transwell **(G)** analysis of migration in PDAC cell lines transfected with pLV-IL37 plasmid. The data are presented as the means ± SD from three independent experiments. *P < 0.05 and **P < 0.01.

Furthermore, we want to investigate whether rhIL-37 can affect sensitivity of PDAC cells to Gem (2 μM, 24 h) *in vitro*. Treatment of PANC-1, MIA-PaCa2 and SW1990 cells with rhIL-37 and Gem dramatically increased the G1 phase distribution when compared to Gem or IL-37 single treatment (Figure 5A,* P* < 0.05). The combination treatment also significantly increased apoptosis when compared to single treatment, suggesting that IL-37 was able to sensitize anti-tumor efficacy of Gem (Figure 5B,* P* < 0.05).

To test whether IL-37 alone is sufficient to inhibit PDAC cells migration, we used rhIL-37 (100 ng/ml) to treat PANC-1, MIA-PaCa2 and SW1990 cell lines. We then examined the motility and migration of PDAC cells by wound-healing assay and transwell assay. Wound-healing assay (Figure 5C, left) and the transwell data (Figure 5C, right) confirmed that rhIL-37 inhibited the migratory activity of PDAC cell lines compared with control cells (*P* < 0.05). To confirm the rhIL-37 suppression the migration of PDAC cells was not attributed to the decreased proliferation of the cells, we performed the cell proliferation experiment by real-time cell analysis (RTCA) system 24. The data showed that rhIL-37 did not decreased proliferation of the PDAC cell lines at 24 h and 36 h (Figure S2, *P* > 0.05). But rhIL-37 can decrease the PDAC cell lines proliferation at 48 h and more than 48 h (Figure S2, *P* < 0.01).

To further understand the role of endogenous IL-37 in PDAC migration, we induced ectopic IL-37 expression in SW1990 and MIA-PaCa-2 (Figure 5D). The secretion and concentration of IL-37 in culture supernatant with transfected cells were increased (Figure 5E). Motility (Figure 5F) and migration (Figure 5G) of PDAC cells was inhibited after transfected with pLV-IL37 plasmid (*P* < 0.01 and *P* < 0.05). These data indicated that IL-37 sensitized efficacy of Gem on PDAC cells and inhibited PDAC cells migration *in vitro*.

### IL-37 binding to its receptors (IL-18Rα and IL-1R8) inhibited HIF-1α expression by suppressed phosphorylation of STAT3 (705)

**Figure 6:**
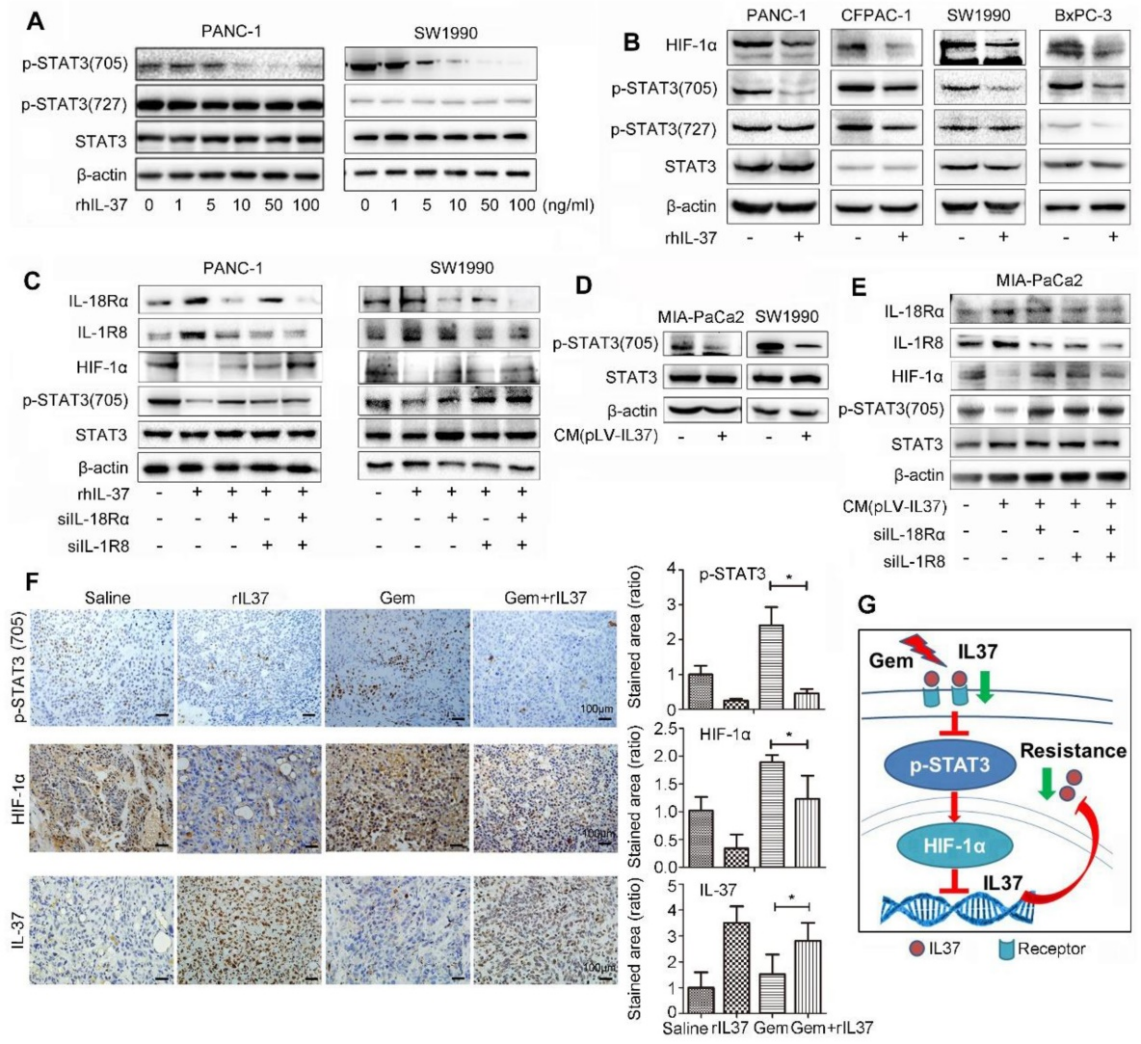
** IL-37 binding to its receptors (IL-18Rα and IL-1R8) inhibited HIF-1α expression by suppressed phosphorylation of STAT3 (705). (A)** Western blot analysis of p-STAT3 (705) and p-STAT3 (727) expression in PANC-1 and SW1990 cells treated with different concentration of rhIL-37 at 1 h. **(B)** Western blot analysis of HIF-1α, p-STAT3 (705) and p-STAT3 (727) expression in PANC-1, CFPAC-1, SW1990 and BxPC-3 PDAC cell lines treated with rhIL-37 (100 ng/ml) at 1 h. **(C)** Western blot analysis of the receptors IL-18Rα, IL-1R8, p-STAT3 (705) and HIF-1α expression in PANC-1 and SW1990 cell lines transfected with siIL-18Rα and siIL-1R8. (D) Western blot analysis of p-STAT3 (705) expression in MIA-PaCa2 and SW1990 cells cultured with conditional medium (pLV-IL-37). **(E)** Western blot analysis of IL-18Rα, IL-1R8, p-STAT3 (705) and HIF-1α expression in MIA-PaCa2 cell line transfected with siIL-18Rα and siIL-1R8 with conditional medium (pLV-IL-37). **(F)** Left: IHC assays of p-STAT3 (705), HIF-1α and IL-37 expression in the mouse PDAC samples with different treatment. Right: the ratio of stained area on p-STAT3 (705), HIF-1α and IL-37 in mouse samples. **(G)** Schematic of the roles of IL-37 in PDAC. *P < 0.05.

IL-37 with different concentration robustly inhibited the levels of p-STAT3 (705), but not p-STAT3 (727) in PANC-1 and SW1990 cell lines (Figure 6A). Furthermore, PANC-1, CFPAC-1, SW1990 and BxPC-3 PDAC cell lines were used to verify the universal suppression phenomenon of IL-37 on p-STAT3 (705) and its down-stream target HIF-1α (Figure 6B). The result was also showed that rhIL-37 can inhibit HIF-1α expression by suppressed STAT3 phosphorylation at 705.

Nold-Petry CA et al reported that IL-37 requires its receptors IL-18Rα and IL-1R8 to carry out the anti-inflammatory program upon innate signal transduction 25. To investigate the role of IL-37 receptor, we knock-down IL-18Rα and IL-1R8 respectively in PANC-1 and SW1990 cell lines (Figure 6C). The depletion of IL-37 receptors was able to mitigate the inhibition of p-STAT3 (705) phosphorylation by IL-37. Furthermore, we repeated the suppression experiments of p-STAT3 with culture supernatant of transfected pLV-IL37 cells. The data showed an obviously inhibition of p-STAT3 (705) expression treated with condition medium (pLV-IL37 cells) (Figure 6D). And the condition medium (pLV-IL37 cells) restored the expression of the p-STAT3 (705) with knock-down IL37 receptors (Figure 6E). Importantly, we performed the IHC assays with the mouse PDAC samples and it showed p-STAT3 (705) and its down-stream HIF-1α expression were extremely inhibited in Gem plus rhIL-37 group compared Gem groups (*P* < 0.05), but IL-37 expression was induced in Gem plus rhIL-37 group compared Gem groups (*P* < 0.05) (Figure 6F). At last, the level of p-STAT3 (705) and HIF-1α expression was measured by IHC in human PDAC samples (n=85). The statistical data showed that there was a positive relationship between HIF-1α and p-STAT3 (705) expression (Figure S3, *P* = 0.023). These data suggested that IL-37 inhibited phosphorylation of STAT3 (705) and HIF-1α expression by binding to its receptors IL-1R8 and IL-18Rα.

Taken together, our data supported that IL-37 loss in PDAC promoted the STAT3 pathway activation and HIF-1α expression, and HIF-1α expression inhibited IL-37 transcription, then IL-37 loss caused Gem resistance and progression (Figure 6G).

## Discussion

IL-37, as an IL-1 family cytokine, has been demonstrated to be an immunosuppressive factor in many inflammatory and autoimmune diseases 1,9,26. Recent studies have reported the antitumor role of IL-37 in various tumor types, such as lung cancer 13,27, colon cancer 28, hepatocellular carcinoma 10,11, renal cancer 29, breast cancer 30, cervical cancer 31 and gallbladder cancer 32 cases. IL-37 inhibits tumor progression through adaptive antitumor immunity and multiple tumor-suppressive signaling pathways. However, the effect of IL-37 on PDAC development and chemo-resistance has not been reported.

In the present study, our data showed that IL-37 expression was markedly associated with tumor progression and chemo-resistance *in vitro* and *in vivo*. The data showed that IL-37 was drastically lower in serum and tumor specimens with PDAC patients than with healthy people and adjacent normal pancreatic tissues. Importantly, reduced IL-37 expression in PDACs was closely associated with clinicopathological features, RFS and OS. Zhang et al 33 and Huo et al 34 reported that elevated serum levels of IL-37 correlate with poor prognosis in gastric cancer and epithelial ovarian cancer patients. The trend of serum IL-37 level in PDAC seems discrepancy in gastric cancer and epithelial ovarian cancer. Firstly, IL-37 level is the total secretion content in serum including not only inflammatory cells secreted IL-37 but also caner-derived IL-37. The serum IL-37 level is not specific for cancer cells. Secondly, the discrepancy may be due to different cancer types with diverse tumor microenvironment, which need further investigations. For example, some related co-factors promoted to gastric cancer development and systematic inflammation such as the infection of Helicobacter pylori (HP) and the EB virus. Inflammations caused by HPV infection played a crucial role in cervical cancer. It has been verified IL-37 has stronger anticancer ability in HPV^+^ Hela cells than in HPV^-^ C33A cells 31. Thirdly, the pathogenesis of different tumors is varied and IL-37 may be a double-edged sword that has multiple roles in different phase and different cancer types. Lastly, our sample size is relatively smaller than gastric cancer and epithelial ovarian cancer patients. Further studies in larger PDAC patient population are necessary to confirm the significance of the serum IL-37 levels.

As the standard treatment for PDAC, Gem has developed resistance in PDAC patients due to desmoplastic stroma and abnormal signaling pathways 35,36. Targeting S1PR1/STAT3 loop can abrogate desmoplasia and chemosensitize PDAC to Gem 37. We, for the first time, demonstrated that IL-37 expression was positively correlated with Gem efficacy and it can sensitize efficacy of Gem *in vitro* and *in vivo*. Further analysis suggested that there was a negative relationship between HIF-1α and IL-37. HIF-1α, as an oncogene, directly bind to the HREs of IL-37 promoter and suppressed the IL-37 transcription activity in PDACs. Importantly, IL-37 can't affect desmoplasia in PDAC.

In the current study, we found strong evidence supporting IL-37 as a tumor suppressor gene. What is the mechanism underlying the inhibition of tumor progression and sensitization efficacy of Gem by IL-37? Extracellular IL-37 binds to the receptor IL-18Rα and IL-1R8 to form the IL-37/IL-18Rα/IL-1R8 tripartite complex. The signaling pathways suppress inflammation by increasing the activity of STAT3 and PTEN. Intracellular IL-37 can bind to p-Smad3 and translocate into the nucleus to regulate the downstream signaling and influence cellular behavior 5. Intracellular mature IL-37 suppresses tumor metastasis via inhibiting Rac1 activation 13. Intracellular IL-37b interacts with Smad3 to suppress multiple signaling pathways and the metastatic phenotype of tumor cells 14. IL-37 inhibits IL-6/STAT3 pathway in non-small cell lung cancer 27, cervical cancer 31 and murine allergic rhinitis 38. To explore the molecular mechanism, we treated PDAC cells with IL-37 stimulation and identified the suppression of IL-37 on STAT3 phosphorylation at tyrosine 705 in PDAC cell lines and the HIF-1α expression, down-stream target of STAT3 pathway, also decreased.

In conclusion, our research uncovered IL-37/STAT3/HIF-1α negative feedback signaling drives tumor development and Gem resistance in PDAC. Therefore, sustaining IL-37 expression not only sensitizes efficacy of Gem but also may be an effective approach to treat PDAC. IL-37 might serve as a promising therapeutic marker and a potential strategy for PDAC treatment.

## Supplementary Material

Supplementary figures and table.

## Figures and Tables

**Table 1:** Correlation of IL-37 expression to clinicopathological features in PDAC

Parameters	IL-37		χ^2^	P	*r*
	-/+	++/+++			
**Age (years)**			0.317	0.665	0.061
<60	23	18			
≥60	22	22			
**Gender**			0.898	0.389	-0.103
Male	26	19			
Female	19	21			
**Histological grade**			26.972	0.000^a^	-0.563
G1, G2	13	34			
G3	32	6			
**Tumor size**			18.378	0.000^ a^	-0.465
T1	6	23			
T2	39	17			
**LN metastasis**			39.178	0.000^a^	-0.679
N0	10	36			
N1	35	4			
**Vessel invasion**			19.552	0.000^a^	-0.480
M0	20	36			
M1	25	4			

NOTE: Statistical data on IL-37 expression in relation to clinicopathological features for surgical PDAC specimens. *P* values were calculated using the chi-square test. Abbreviation: LN, lymph node. ^a^Statistically significant (*P* < 0.05)

**Table 2:** Univariate and multivariate analysis of clinicopathological factors for overall survival (OS) and relapse-free survival (RFS)

Variables	OS		RFS	
	HR (95.0% CI)	*P*	HR (95.0% CI)	*P*
**UNIVARIATE ANALYSIS**				
Age	0.986 (0.596-1.631)	0.957	0.875 (0.528-1.450)	0.605
Gender	1.093 (0.661-1.808)	0.728	1.045 (0.632-1.728)	0.865
Tumor size	2.391 (1.342-4.262)	0.003^a^	2.356 (1.347-4.121)	0.003^a^
Vessel invasion	4.770 (2.736-8.316)	0.000^a^	7.814 (4.188-14.576)	0.000^a^
LN metastasis	5.507 (3.074-9.867)	0.000^a^	8.861 (4.712-16.664)	0.003^a^
Grade	3.464 (2.031-5.909)	0.000^a^	3.417 (2.003-5.830)	0.000^a^
IL-37	0.118 (0.061-0.228)	0.000^a^	0.054 (0.022-0.130)	0.000^a^
**MULTIVARIATE ANALYSIS**				
Tumor size	0.751 (0.336-1.679)	0.485	1.326 (0. 666-2.642)	0.422
Vessel invasion	2.285 (1.222-4.271)	0.010^a^	6.697 (3.187-14.076)	0.000^a^
LN metastasis	2.562 (1.267-5.181)	0.009^a^	5.435 (2.428-12.167)	0.000^ a^
Grade	2.050 (1.147-3.665)	0.015^a^	1.089 (0.584-2.031)	0.789
IL-37	0.332 (0.121-0.910)	0.032^a^	0.212 (0.066-0.676)	0.009^a^

NOTE: Univariate analysis: log rank; multivariate Cox proportional hazards analysis. Abbreviations: HR, hazard ratio; CI, confidence interval; LN, lymph node. ^a^Statistically significant (*P* < 0.05).
