# Supplementary material for: IL-37/ STAT3/ HIF-1α negative feedback signaling drives gemcitabine resistance in pancreatic cancer
Source: Theranostics. 2020 Mar 4;10(9):4088–100. doi: 10.7150/thno.42416 (PMC7086367; doi:10.7150/thno.42416)
Supplement: Supplementary file 1 — Supplementary figures and table. [file thnov10p4088s1.pdf]

Supplementary materials

Figure S1. Examined IL-37 and α-SMA expression in PDAC samples by IHC and performed the Masson stain (n=85).

And the statistical data showed that IL-37 was not associated with α-SMA expression and stromal fibrosis ($P > 0.05$).

Figure S2. Measurement the effect of rIL-37 on proliferation of the PDAC cell lines by real-time cell analysis (RTCA) system.

The data showed that rhIL-37 (100 ng/ml) did not decreased proliferation of the PDAC cell lines (SW1990, PANC-1 and MIA-PaCa2) at 24 h and 36 h ($P > 0.05$). But rhIL-37 can decrease proliferation of the PDAC cell lines at 48 h and more than 48 h ($P < 0.01$). **$P < 0.01$ and ***$P < 0.01$.

Figure S3. Examination the level of p-STAT3 (705) and HIF-1α in PDAC samples by IHC (n=85).

The statistical data showed that there was a positive relationship between HIF-1α and p-STAT3 (705) expression ($P = 0.023$).

Figure S1

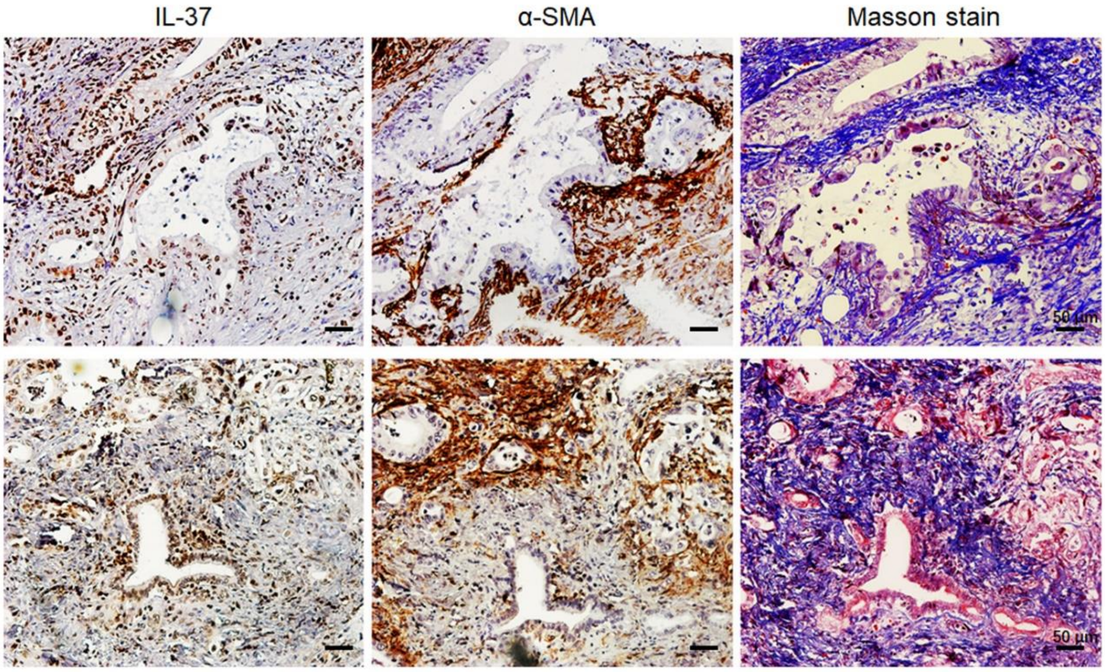

IL-37

α-SMA	-/++	++/+++	r	*P*
-/+	35	8	0.035	0.748
++/+++	33	9		

IL-37

Masson	-/++	++/+++	r	*P*
-/+	60	12	0.196	0.072
++/+++	8	5		

Figure S2

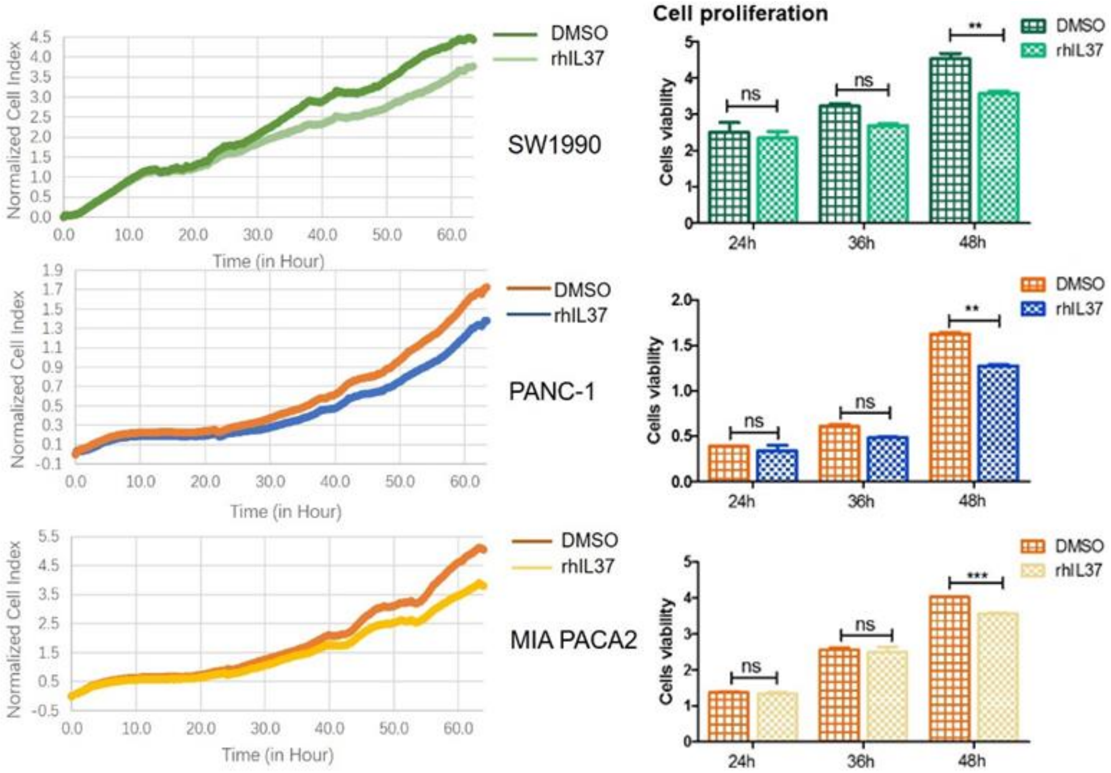
Cell proliferation
DMSO
rhIL37
SW1990
PANC-1
MIA PACA2
Normalized Cell Index
Time (in Hour)
Cells viability
ns
**
***
24h
36h
48h

Figure S3

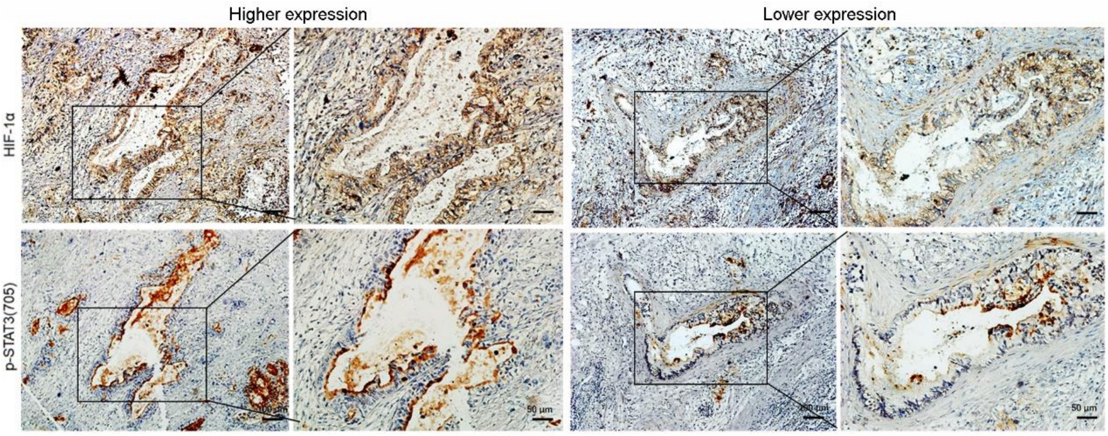

p-STAT3 (705)

HIF-1α		-/++	++/+++	r	*P*
	-/+	46	12	0.246	0.023
	++/+++	15	12		

Table S1 Antibodies and primers

**Antibodies**				
Name	Manufacturer	Number	Type	Usage
IL-37	Abcam	ab153889	Monoclonal	IHC
IL-37	Abcam	ab 57187	Monoclonal	WB, IF
SIGIRR	Abcam	ab 25875	Polyclonal	WB,IHC
IL18R1	Abcam	ab117432	Polyclonal	WB,IHC
HIF-1α	Abcam	ab113642	Monoclonal	WB,IHC
Stat3	Cell Signaling Technology	D3Z2G	Monoclonal	WB
Phospho-Stat3 (Tyr705)	Cell Signaling Technology	D3A7	Monoclonal	WB
Phospho-Stat3 (Ser727)	Cell Signaling Technology	D8C2Z	Monoclonal	WB
β-actin	Cell Signaling Technology	8H10D10	Monoclonal	WB
DAPI	Beyotine Biotechnology	C1002		IF
**siRNAs**	**sequences (5'-3')**			
IL37 #1	UCCUGGAACAUGCUGGAGUTT/ ACUCCAGCAUGUUCCAGGATT			
IL37 #2	UGGGGUGAVAGAUAAAUUUTT/ AAAUUUAUCUGUCACCCCATT			
SIGIRR #1	GCAGCUCAGUGGCUCUGAATT/ UUCAGAGCCACUGAGCUGCTT			
SIGIRR #2	GCCGCCCUGCUCUAUGUCATT/ UGACAUAGAGCAGGGCGGCTT			
IL18R1 #1	CCAGGCCACGUCUUCACAATT/ UUGUGAAGACGUGGCCUGGTT			
IL18R1 #2	CCUGUUCUUUCCGAGUCUUTT/ AAGACUCGGAAAGAACAGGTT			
HIF-1α #1	GCCGAGGAAGAACUAUGAATT/ UUCAUAGUUCUUCCUCGGCTT			
HIF-1α #2	GCCGCUCAAUUUAUGAAUATT/ UAUUCAUAAAUUGAGCGGCTT			
Positive control	UGACCUCAACUACAUGGUUTT/AACCAUGUAGUUGAGGUCATT			
Negative control	UUCUCCGAACGUGUCACGUTT/ACGUGACACGUUCGGAGAATT			
**Primer sequences (5'-3')**				
IL37	F:CCTGGACTCTGGGAATCTCA R: AGAGGCTGAGCTCAAGGATG			
HIF-1α	F:GGTGCTGATTTGTGAACCCA R: TATCCAGGCTGTGTCGACTG			
GAPDH	F:GGAGCGAGATCCCTCCAAAATR:GGCTGTTGTCATACTTCTCATGG			

IL-37-1-P*	F: TGTTGAAAGCTCTCGGTCAA	R: TGCTCTGCAACCTCTTTTCA
IL-37-2-P*	F: TTCAGGGATACTGCCAAACC	R: AGGGCTTCAGGACTCAACAA
IL-37-3/4-P*	F: CCACGTGAAGCACATCTGAA	R: CCAGCAATGCCCTACTTGTT

WB: Western boltting; IHC: Immunohistochemistry; IF:Immunofluorescence; siRNA: Small interfering RNA; P*: Promoter
